# Supplementary material for: Factors associated with the intention to vaccinate and price sensitivity to the human papillomavirus (HPV) vaccine among Chinese male medical college students: a cross-sectional survey
Source: PeerJ. 2025 Jun 30;13:e19699. doi: 10.7717/peerj.19699 (PMC12273698; doi:10.7717/peerj.19699)
Supplement: Supplemental Information 1 [file peerj-13-19699-s001.pdf]

STROBE Statement—Checklist of items that should be included in reports of ***cross-sectional studies***

	Item No	Recommendation	Location in Manuscript (Page / Line No.)
**Title and abstract**	1	(*a*) Indicate the study's design with a commonly used term in the title or the abstract	**Lines 26-30**
		(*b*) Provide in the abstract an informative and balanced summary of what was done and what was found	**Lines 31-44**
**Introduction**			
Background/rationale	2	Explain the scientific background and rationale for the investigation being reported	**Lines 47-97**
Objectives	3	State specific objectives, including any prespecified hypotheses	**Lines 98-103**
**Methods**			
Study design	4	Present key elements of study design early in the paper	**Lines 107-110**
Setting	5	Describe the setting, locations, and relevant dates, including periods of recruitment, exposure, follow-up, and data collection	**Lines 107-110**
Participants	6	(*a*) Give the eligibility criteria, and the sources and methods of selection of participants	**Lines 107-119**
Variables	7	Clearly define all outcomes, exposures, predictors, potential confounders, and effect modifiers. Give diagnostic criteria, if applicable	**Lines 126-157**
Data sources/ measurement	8*	For each variable of interest, give sources of data and details of methods of assessment (measurement). Describe comparability of assessment methods if there is more than one group	**Lines126-157**
Bias	9	Describe any efforts to address potential sources of bias	**Lines 110-114**<br>**Lines 121-125**
Study size	10	Explain how the study size was arrived at	**Lines 115-119**
Quantitative variables	11	Explain how quantitative variables were handled in the analyses. If applicable, describe which groupings were chosen and why	**Lines 133-157**
Statistical methods	12	(*a*) Describe all statistical methods, including those used to control for confounding	**Lines 158-165**
		(*b*) Describe any methods used to examine subgroups and interactions	**Lines 158-165**
		(*c*) Explain how missing data were addressed	**Lines 110-112**
		(*d*) If applicable, describe analytical methods taking account of sampling strategy	**Line 108**
		(*e*) Describe any sensitivity analyses	**-**
**Results**			
Participants	13*	(a) Report numbers of individuals at each stage of study—eg numbers potentially eligible, examined for eligibility, confirmed eligible, included in the study, completing follow-up, and analysed	**Lines 115-119**<br>**Lines 211-216**
		(b) Give reasons for non-participation at each stage	**Lines 115-119**

			**Lines 211-216**
		(c) Consider use of a flow diagram	**Lines 115-119**<br>**Lines 211-216**
Descriptive data	14*	(a) Give characteristics of study participants (eg demographic, clinical, social) and information on exposures and potential confounders	**Table 1-2**
		(b) Indicate number of participants with missing data for each variable of interest	**Lines 211-216**
Outcome data	15*	Report numbers of outcome events or summary measures	**Lines 169-184**
Main results	16	(*a*) Give unadjusted estimates and, if applicable, confounder-adjusted estimates and their precision (eg, 95% confidence interval). Make clear which confounders were adjusted for and why they were included	**Table 3-4**<br>**Figure 1-2**<br>**Lines 186-209**
		(*b*) Report category boundaries when continuous variables were categorized	**Lines 182-184**
		(*c*) If relevant, consider translating estimates of relative risk into absolute risk for a meaningful time period	**-**
Other analyses	17	Report other analyses done—eg analyses of subgroups and interactions, and sensitivity analyses	**Lines 210-237**
**Discussion**			
Key results	18	Summarise key results with reference to study objectives	**Lines 240-244**<br>**Lines 255-259**<br>**Lines 295-296**<br>**Lines 312-313**<br>**Lines 316-323**
Limitations	19	Discuss limitations of the study, taking into account sources of potential bias or imprecision. Discuss both direction and magnitude of any potential bias	**Lines 337-360**
Interpretation	20	Give a cautious overall interpretation of results considering objectives, limitations, multiplicity of analyses, results from similar studies, and other relevant evidence	**Lines 244-360**
Generalisability	21	Discuss the generalisability (external validity) of the study results	**Lines 337-341**<br>**Lines 343-345**<br>**Lines 352-357**
**Other information**			
Funding	22	Give the source of funding and the role of the funders for the present study and, if applicable, for the original study on which the present article is based	**Lines 402-403**

*Give information separately for exposed and unexposed groups.

Note: An Explanation and Elaboration article discusses each checklist item and gives methodological background and published examples of transparent reporting. The STROBE checklist is best used in conjunction with this article (freely available on the Web sites of PLoS Medicine at http://www.plosmedicine.org/, Annals of Internal Medicine at http://www.annals.org/, and Epidemiology at http://www.epidem.com/). Information on the STROBE Initiative is available at www.strobe-statement.org.
